# Microbiome-mediated regulation of pathogen virulence: Molecular mechanisms and clinical implications

**DOI:** 10.1080/21505594.2026.2721749

**Published:** 2026-08-25

**Authors:** Ahmed J. Alzahrani

**Affiliations:** a College of Medicine, Al-Imam Mohammed Ibn Saud Islamic University, Riyadh, Saudi Arabia; b Laboratory Department, Dr. Sulaiman Al Habib Medical Group, Riyadh, Saudi Arabia

**Keywords:** Microbiome, pathogen virulence, quorum sensing, metabolites, probiotics, host-pathogen interactions, antimicrobial resistance

## Abstract

The human microbiome profoundly influences pathogen virulence and disease susceptibility through diverse molecular mechanisms. This review, focused on literature from 2016 to 2026, synthesizes knowledge on microbiome-mediated regulation examining major pathogens including *Vibrio cholerae*, enterohemorrhagic *Escherichia coli*, *Salmonella* species, *Clostridioides difficile*, and *Staphylococcus aureus*. A triangular interaction model is presented, linking host immunity, resident microbiome, and invading pathogen to frame infection outcomes. Commensal bacteria regulate pathogen behavior through quorum sensing interference, metabolite-mediated regulation via short-chain fatty acids and bile acids, competitive exclusion, immune response modulation, and direct antimicrobial production. Key species—*Lactobacillus* spp. *Bifidobacterium* spp. *Bacteroides thetaiotaomicron*, and *Faecalibacterium prausnitzii*—employ distinct molecular strategies against these pathogens. Organ-on-chip platforms and multi-omics advances reveal context-dependent interactions. Clinical applications including rationally designed probiotics, fecal microbiota transplantation, and precision microbiome engineering show promise despite challenges of inter-individual microbiome variability, representing a paradigm shift toward ecosystem-based infectious disease management.

## Introduction

The human body harbors trillions of microorganisms collectively known as the microbiome, which plays fundamental roles in host physiology, metabolism, and immune function. The recognition that commensal microorganisms influence infectious disease outcomes has deep historical roots. Early observations by Metchnikoff in the early 20th century suggested that lactic acid bacteria could promote health and longevity, while mid-century studies demonstrated that antibiotic disruption of the gut microbiota increased susceptibility to enteric infections [1]. The molecular era of microbiome research, beginning in the 1990 s with culture-independent sequencing methods, revealed the extraordinary complexity and functional capacity of human-associated microbial communities [2]. By the early 2000 s, metagenomic surveys had established that the human gut microbiome comprises hundreds of bacterial species with collective gene content exceeding that of the human genome by orders of magnitude [3].

Building on this foundation, research from approximately 2010 to 2015 demonstrated that microbiome composition correlates with susceptibility to specific pathogens and that commensals can directly interfere with pathogen colonization and virulence factor expression [4]. These discoveries set the stage for mechanistic investigations into how resident microbiota regulate pathogen behavior at the molecular level. While the microbiome’s importance in maintaining health has been recognized for decades, advances since 2016 have revealed its critical role in modulating pathogen virulence and infection outcomes [5–7]. This review focuses on literature published between 2016 and 2026, a period marked by the application of organ-on-chip platforms, high-resolution multi-omics, and large-scale metagenomic analyses that have moved the field from correlation to causation. This paradigm shift from viewing pathogens in isolation to understanding them within the context of complex microbial communities has profound implications for infectious disease management.

Global metagenomic surveys have documented substantial interpersonal variation in gut microbiome composition and diversity. Analysis of over 12,000 gut metagenomes from diverse populations reveals that taxonomic and functional diversity varies widely among individuals and correlates with colonization resistance to opportunistic pathogens such as Enterobacteriaceae [7]. Human challenge studies and cohort analyses demonstrate that individuals with higher baseline microbiome diversity and specific commensal taxa exhibit reduced susceptibility to *Vibrio cholerae* infection, with interpersonal microbiome differences predicting clinical outcomes [8]. Experimentally, synthetic human gut communities comprising up to 50 defined species show that higher community diversity increases colonization resistance to *Klebsiella pneumoniae* and *Salmonella enterica* serovar Typhimurium through collective nutrient consumption and niche occupation [6]. These findings underscore that microbiome-mediated pathogen regulation is a community-level phenomenon shaped by ecological interactions among hundreds of microbial species.

The concept of microbiome-mediated pathogen regulation emerged from observations that individuals with similar pathogen exposures experience vastly different infection outcomes. Epidemiological studies have demonstrated that microbiome composition correlates with susceptibility to various infections, from gastrointestinal pathogens like *Vibrio cholerae* and enterohemorrhagic *Escherichia coli* (EHEC) to respiratory pathogens [9; 10]. These observations prompted investigations into the molecular mechanisms by which commensal microorganisms influence pathogen behavior.

Commensal bacteria employ multiple strategies to regulate pathogen virulence. These include interference with bacterial communication systems (quorum sensing), production of metabolites that modulate virulence gene expression, competition for nutrients and colonization sites, modulation of host immune responses, and direct antimicrobial activity. Understanding these mechanisms is crucial for developing novel therapeutic approaches that harness the protective capacity of the microbiome.

This review synthesizes current knowledge on microbiome-mediated regulation of pathogen virulence, focusing on molecular mechanisms and clinical applications. We examine how commensal bacteria interfere with pathogen behavior through multiple pathways, discuss recent technological advances that have enabled mechanistic insights, and explore the therapeutic potential of microbiome-based interventions. Throughout, we emphasize species-specific mechanisms employed by key beneficial bacteria—*Lactobacillus* spp., *Bifidobacterium* spp., *Bacteroides thetaiotaomicron*, and *Faecalibacterium prausnitzii*—against major pathogens including *Vibrio cholerae*, EHEC, *Salmonella* spp., *Clostridioides difficile*, and *Staphylococcus aureus*.

## The triangular interaction model: host, microbiome, and pathogen

**Figure 1.**
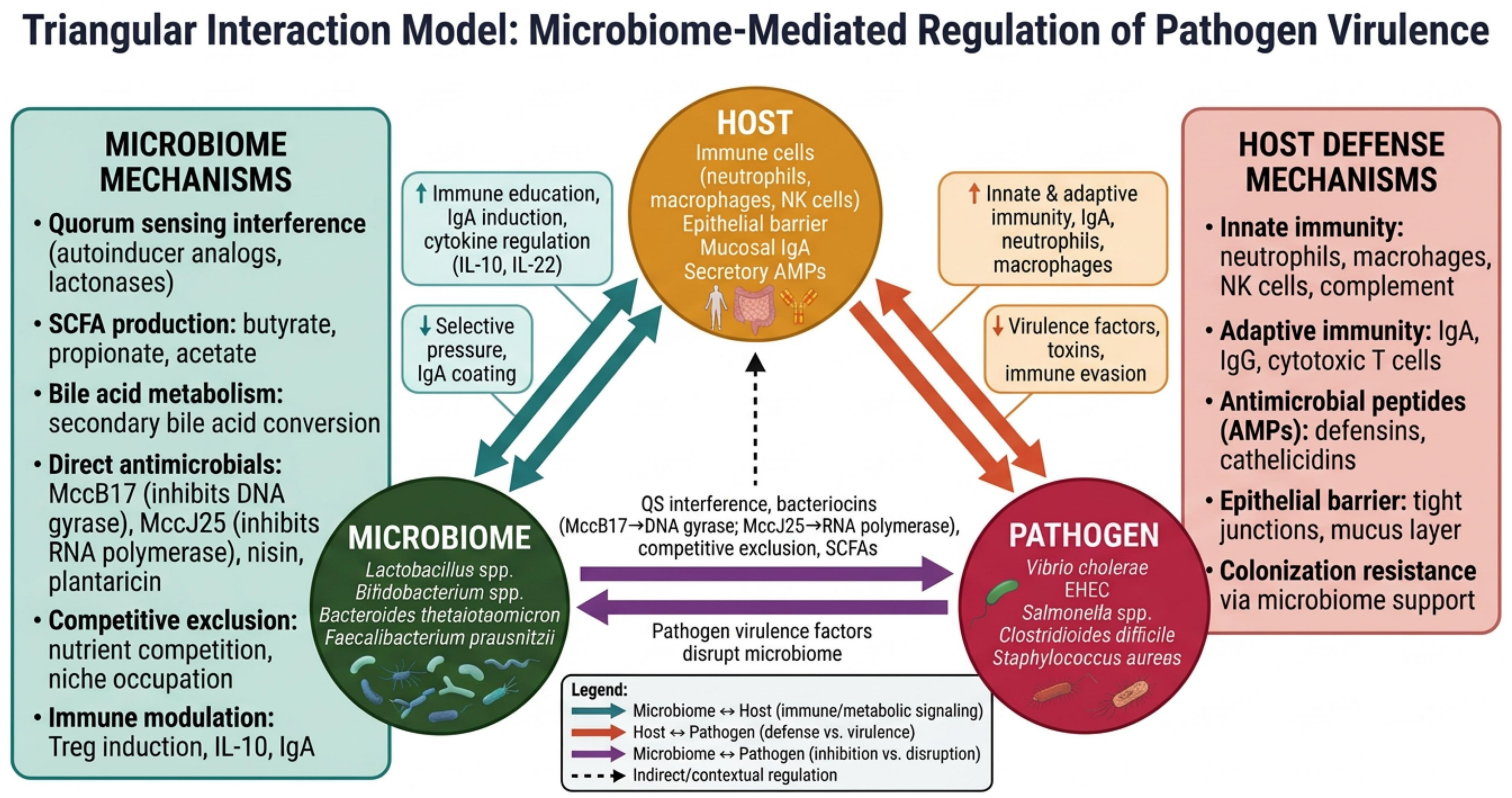
The triangular interaction model of host-microbiome-pathogen dynamics.

Pathogen virulence and infection outcomes are determined by a triangular interaction model involving three interconnected components: the host immune system, the resident microbiome, and the invading pathogen (Figure 1). In this model, each vertex influences the other two through bidirectional molecular crosstalk. The host immune system shapes microbiome composition through secretory IgA, antimicrobial peptides, and inflammatory mediators, while the microbiome educates and modulates host immunity through metabolites, microbial-associated molecular patterns (MAMPs), and direct immune cell interactions. Simultaneously, the microbiome directly regulates pathogen behavior via quorum sensing interference, metabolite production, nutrient competition, and antimicrobial secretion, while pathogens can exploit or disrupt commensal communities to facilitate colonization. Finally, the host immune response targets the pathogen through innate and adaptive mechanisms, and pathogens deploy virulence factors to evade or subvert host defenses. Critically, microbiome-mediated effects on pathogen virulence are context-dependent, varying with community composition, host immune status, pathogen strain, and environmental factors such as diet and antibiotic exposure. This triangular framework emphasizes that infection outcomes cannot be understood by studying host-pathogen interactions in isolation; rather, the resident microbiome is an integral determinant of virulence regulation and disease susceptibility [4,5].

Throughout the remainder of this review, we refer to this triangular interaction model when discussing specific mechanisms and clinical applications, recognizing that each mechanism operates within this complex three-way interplay.

### Quorum sensing and inter-kingdom communication

Quorum sensing (QS) is a cell density-dependent communication system that bacteria use to coordinate collective behaviors, including virulence factor production, biofilm formation, and metabolic adaptation. Pathogens such as *Pseudomonas aeruginosa*, *Vibrio cholerae*, and enterohemorrhagic *E. coli* rely on QS to regulate virulence in response to population density and environmental cues [11].

The microbiome can interfere with pathogen QS through multiple mechanisms. Commensal bacteria produce molecules that mimic or antagonize pathogen autoinducers, effectively disrupting pathogen communication networks. For example, certain *Lactobacillus* species produce compounds that interfere with acyl-homoserine lactone (AHL)-based QS systems used by Gram-negative pathogens [12]. Additionally, some commensals express enzymes that degrade QS signals, a process termed quorum quenching. These enzymes, including AHL-lactonases and AHL-acylases, hydrolyze autoinducer molecules before they can activate pathogen virulence programs [13].

Commensal bacteria also engage in “eavesdropping” on pathogen signaling molecules, a phenomenon in which resident microbiota detect and respond to pathogen-derived QS signals without producing those signals themselves [14]. This inter-species and inter-kingdom communication allows commensals to sense the presence of pathogens and mount competitive or defensive responses, such as increased antimicrobial production or enhanced colonization of mucosal niches. Conversely, pathogens may eavesdrop on commensal signaling to time virulence factor expression or identify favorable colonization sites. These bidirectional eavesdropping interactions add an additional layer of complexity to the triangular interaction model, highlighting that chemical communication networks extend across the entire microbial community and influence infection dynamics [11,14].

Beyond bacteria-bacteria communication, inter-kingdom signaling between bacteria and host cells, or between bacteria and fungi, adds further complexity. Probiotic yeast metabolites have been shown to inhibit bacterial virulence and QS systems, demonstrating that eukaryotic microbiome members also participate in pathogen regulation [12]. The host itself produces molecules that can modulate bacterial QS, creating a complex network of chemical signals that collectively determine pathogen behavior [15].

### Technological advances in studying microbiome-pathogen interactions

Recent technological advances have transformed our ability to study microbiome-pathogen interactions with unprecedented resolution and physiological relevance. Organ-on-chip platforms, which integrate human intestinal epithelial cells, immune cells, and complex microbial communities in microfluidic devices, now enable real-time observation of host-microbiome-pathogen dynamics under controlled conditions [16; 17]. These systems recapitulate key features of the intestinal environment, including peristalsis-like fluid flow, oxygen gradients, and mucus production, allowing researchers to test how specific commensal strains protect against pathogen-induced epithelial damage and inflammation. For example, colon-on-chip models have demonstrated that defined commensal consortia can prevent *Salmonella*-induced epithelial barrier disruption and reduce inflammatory cytokine secretion, providing causal evidence for protective mechanisms identified in correlative human studies [16].

In parallel, multi-omics approaches – integrating metagenomics, metatranscriptomics, metaproteomics, and metabolomics – have revealed the functional dynamics of microbiome-pathogen interactions in clinical cohorts and experimental models. Large-scale metagenomic analyses of over 12,000 human gut microbiomes have identified specific microbial functions, including short-chain fatty acid production, iron metabolism, and quorum-sensing-related pathways, that correlate with colonization resistance to Enterobacteriaceae across diverse populations [7]. Metatranscriptomic studies further show that pathogen virulence gene expression is dynamically regulated by the surrounding microbial community, with commensal-derived metabolites directly modulating pathogen transcriptional programs. These high-resolution datasets enable systems-level modeling of microbiome-pathogen interactions and support the rational design of therapeutic microbial consortia tailored to specific pathogens and host contexts [18].

Together, organ-on-chip platforms and multi-omics technologies have shifted the field from descriptive correlation to mechanistic causation, providing the tools necessary to dissect species-specific and context-dependent effects of the microbiome on pathogen virulence.

## Molecular mechanisms of microbiome-mediated virulence regulation

The microbiome employs diverse molecular strategies to regulate pathogen virulence. These mechanisms operate within the triangular interaction model described in Section 2, with each mechanism involving direct microbiome-pathogen interactions, indirect effects mediated by host responses, or both. This section examines five major mechanisms: quorum sensing interference, metabolite-mediated regulation, competitive exclusion, immune modulation, and direct antimicrobial production. We then discuss species-specific mechanisms employed by key beneficial bacteria.

### Quorum sensing interference

Quorum sensing interference represents a sophisticated mechanism by which commensal bacteria disrupt pathogen communication and virulence coordination. This interference occurs through multiple pathways, each targeting different components of QS systems.

#### Signal mimicry and antagonism

Commensal bacteria produce structural analogs of pathogen autoinducers that compete for binding to QS receptors without activating downstream virulence programs. These antagonistic molecules effectively “jam” pathogen communication, preventing the coordinated expression of virulence factors even when pathogen population density is high. *Lactobacillus* species, for example, produce compounds that interfere with AHL-based QS in *Pseudomonas aeruginosa*, reducing production of virulence factors such as pyocyanin and elastase [12].

#### Enzymatic signal degradation

Quorum quenching enzymes produced by commensals actively degrade QS signal molecules, reducing their effective concentration below the threshold required for virulence activation. AHL-lactonases hydrolyze the lactone ring of AHL molecules, while AHL-acylases cleave the amide bond, both rendering the signals inactive. These enzymes are produced by diverse commensal taxa, including *Bacillus*, *Rhodococcus*, and certain *Bacteroides* species, providing broad-spectrum interference with Gram-negative pathogen QS [13].

#### Receptor-level interference

Some commensal-derived molecules directly interfere with QS receptor function, preventing signal transduction even in the presence of authentic autoinducers. This mechanism provides robust inhibition of virulence programs and has been demonstrated in studies of probiotic effects on *Staphylococcus aureus*, where commensal metabolites reduce expression of the *agr* QS system and associated virulence factors [19].

### Metabolite-mediated regulation

Microbial metabolites serve as key mediators of pathogen virulence regulation, acting through direct effects on pathogen gene expression and indirect effects on the host environment.

#### Short-chain fatty acids (SCFAs)

SCFAs – primarily acetate, propionate, and butyrate – are produced by commensal fermentation of dietary fiber and represent major metabolic outputs of the gut microbiome. These metabolites directly influence pathogen virulence through multiple mechanisms. Butyrate, for example, downregulates virulence gene expression in enterohemorrhagic *E. coli* by modulating intracellular pH and affecting the activity of virulence regulators. SCFAs also strengthen intestinal barrier function, reducing pathogen translocation, and modulate host immune responses to favor protective immunity [20].

The effects of SCFAs are pathogen-specific and context-dependent. While butyrate generally suppresses virulence in EHEC, it can enhance virulence in *Salmonella* under certain conditions by serving as a metabolic signal indicating the intestinal environment. This context-dependency underscores the complexity of metabolite-mediated regulation and the importance of understanding species-specific responses.

#### Bile acids

Bile acids, which are produced by the host liver and extensively modified by gut bacteria, profoundly influence pathogen behavior. Primary bile acids are converted to secondary bile acids by commensal bacteria expressing bile salt hydrolases and 7α-dehydroxylases. These secondary bile acids can either promote or inhibit pathogen virulence depending on the pathogen and specific bile acid species.

*Clostridioides difficile* provides a well-studied example of bile acid-mediated virulence regulation. Primary bile acids such as taurocholate promote *C. difficile* spore germination and vegetative growth, while certain secondary bile acids inhibit germination and toxin production. Microbiome disruption by antibiotics reduces secondary bile acid production, creating a permissive environment for *C. difficile* colonization and disease [21].

#### Other metabolites

Beyond SCFAs and bile acids, the microbiome produces numerous other metabolites that influence pathogen virulence. These include indole derivatives, which modulate virulence gene expression in *E. coli* and *Salmonella*; succinate, which can serve as a respiratory electron acceptor for pathogens during inflammation; and various amino acid metabolites that affect pathogen metabolism and virulence factor production [20].

### Competitive exclusion

Competitive exclusion encompasses multiple mechanisms by which commensals prevent pathogen colonization and expansion through direct competition for resources and niches.

#### Nutrient competition

Commensals and pathogens often compete for the same nutrients, and the outcome of this competition can determine infection success. The microbiome collectively consumes nutrients that pathogens require for growth, creating a nutrient-depleted environment that resists colonization. This mechanism is particularly important for pathogens that require specific nutrients or metabolic niches.

*Salmonella* and *Citrobacter rodentium* exploit inflammation-derived nutrients, particularly tetrathionate and nitrate, which serve as respiratory electron acceptors that provide a growth advantage over fermenting commensals. However, a diverse microbiome can limit pathogen access to these nutrients through rapid consumption or by preventing the inflammatory conditions that generate them [5].

#### Niche occupation

Physical colonization sites in the intestine are limited, and commensals that occupy these niches prevent pathogen attachment and colonization. Mucus-associated bacteria, for example, form dense communities in the mucus layer that physically exclude pathogens from accessing the epithelium. *Bacteroides thetaiotaomicron* and other mucus-degrading commensals maintain close association with the intestinal epithelium, occupying niches that pathogens might otherwise exploit [4].

#### Metabolic cross-feeding networks

The microbiome forms complex metabolic networks in which the products of one species serve as substrates for others. These cross-feeding relationships create stable community structures that resist invasion by pathogens. Disruption of these networks, such as by antibiotics, can create metabolic niches that pathogens exploit for colonization [20].

### Modulation of host immune responses

The microbiome profoundly influences host immune function, and these immunomodulatory effects indirectly regulate pathogen virulence by altering the host environment and immune responses to infection.

#### Immune system education and homeostasis

Commensals educate the developing immune system, promoting the differentiation of regulatory T cells (Tregs) and the production of secretory IgA. These immune components maintain intestinal homeostasis and provide protection against pathogens without causing excessive inflammation. Microbiome-derived metabolites, particularly SCFAs, promote Treg differentiation and function, creating an immune environment that tolerates commensals while remaining responsive to pathogens [22].

#### Barrier function enhancement

The microbiome strengthens intestinal barrier function through multiple mechanisms. Commensal bacteria stimulate mucus production by goblet cells, enhance tight junction integrity between epithelial cells, and promote epithelial cell turnover and repair. These effects reduce pathogen access to the epithelium and limit translocation across the intestinal barrier. Specific commensals, such as *Akkermansia muciniphila*, are particularly effective at enhancing barrier function through direct interactions with epithelial and immune cells [20].

#### Inflammatory response modulation

During infection, the microbiome modulates the intensity and character of inflammatory responses. Excessive inflammation can cause tissue damage and create metabolic niches that favor pathogen expansion, while insufficient inflammation fails to control pathogen growth. The microbiome helps calibrate inflammatory responses to achieve pathogen control while minimizing collateral damage. This calibration occurs through multiple pathways, including production of anti-inflammatory metabolites, stimulation of regulatory immune cells, and competition with pathogens for inflammatory-derived nutrients [22].

### Direct antimicrobial production

Commensal bacteria produce a diverse array of antimicrobial compounds that directly inhibit pathogen growth and virulence. These antimicrobials provide immediate, localized defense against invading pathogens.

#### Bacteriocins

Bacteriocins are ribosomally synthesized antimicrobial peptides that target specific bacterial species or groups. They are produced by many commensal bacteria, particularly lactic acid bacteria such as *Lactobacillus* and *Enterococcus* species. Bacteriocins typically target closely related bacteria by disrupting cell membrane integrity or interfering with cell wall synthesis. The narrow spectrum of bacteriocin activity allows them to target pathogens while sparing phylogenetically distant commensals, maintaining microbiome diversity while providing pathogen defense [23].

#### Microcins

Microcins are small, post-translationally modified antimicrobial peptides produced primarily by Enterobacteriaceae. Unlike bacteriocins, microcins often target intracellular processes rather than membrane integrity. For example, microcin B17 (MccB17) inhibits DNA gyrase, while microcin J25 (MccJ25) inhibits RNA polymerase, thereby blocking nucleic acid synthesis in susceptible bacteria [24]. Microcin-producing commensals provide colonization resistance against pathogenic Enterobacteriaceae, and loss of these producers during antibiotic treatment can increase susceptibility to enteric infections.

#### Hydrogen peroxide and organic acids

Many commensals produce hydrogen peroxide and organic acids (lactic acid, acetic acid) as metabolic byproducts. These compounds create an acidic, oxidative environment that inhibits pathogen growth. *Lactobacillus* species are particularly effective producers of lactic acid, which lowers local pH and directly inhibits acid-sensitive pathogens. The combination of low pH and organic acids provides broad-spectrum antimicrobial activity while being tolerated by acid-resistant commensals [23].

#### Other antimicrobial compounds

Commensals produce numerous other antimicrobial compounds, including biosurfactants, siderophores that sequester iron from pathogens, and enzymes that degrade pathogen virulence factors. The diversity of antimicrobial mechanisms employed by the microbiome provides robust, multi-layered defense against pathogen colonization and virulence.

#### Protection of co-existing commensals

A critical question in understanding direct antimicrobial production is how beneficial bacteria protect themselves and co-existing commensals from their own antimicrobial compounds while still targeting pathogens. Several mechanisms contribute to this selective protection:

*Immunity Proteins*: Many bacteriocin- and microcin-producing bacteria co-express immunity proteins that specifically bind to and neutralize their own antimicrobial peptides, protecting the producer cell from self-toxicity. These immunity proteins are often encoded in the same operon as the antimicrobial gene, ensuring coordinated expression. Closely related commensals may also possess compatible immunity genes, allowing them to coexist with antimicrobial producers [24].

*Spatial Segregation*: In the complex, structured environment of the intestinal mucosa and lumen, spatial segregation limits exposure of sensitive commensals to antimicrobial compounds. Antimicrobial-producing bacteria may occupy specific niches (e.g. mucus layer, epithelial surface) while sensitive commensals reside in different locations (e.g. lumen, crypts), reducing direct contact and antimicrobial exposure.

*Intrinsic Resistance Mechanisms*: Commensals that coexist with antimicrobial producers often possess intrinsic resistance mechanisms, such as efflux pumps, modified cell wall structures, or alternative metabolic pathways that bypass antimicrobial targets. For example, bacteria resistant to microcins may express efflux systems that export the peptides before they reach intracellular targets, or possess mutations in target enzymes (e.g. DNA gyrase, RNA polymerase) that reduce antimicrobial binding [24].

*Metabolic Cross-Feeding and Cooperation*: Antimicrobial-producing bacteria may engage in metabolic cross-feeding with neighboring commensals, providing essential nutrients or metabolites that support their growth and survival despite antimicrobial exposure. This cooperative relationship can stabilize mixed communities and ensure that antimicrobial production targets pathogens without eliminating beneficial neighbors. Experimental studies with *Lactobacillus* and *Bifidobacterium* cell-free extracts have shown that secreted metabolites can stimulate growth of co-existing commensals in a concentration-dependent manner, suggesting that producer strains actively support their neighbors [25].

*Specificity of Antimicrobial Compounds*: Many bacteriocins and microcins exhibit narrow target specificity, inhibiting only closely related species or strains while sparing phylogenetically distant commensals. This specificity arises from the requirement for specific receptors or uptake systems on target cells, which are absent in non-target commensals. As a result, antimicrobial-producing commensals can selectively target invading pathogens (often closely related to the producer) without disrupting the broader microbial community [23].

These protective mechanisms ensure that direct antimicrobial production by commensals contributes to colonization resistance against pathogens while maintaining the stability and diversity of the resident microbiome. Understanding these mechanisms is critical for designing probiotic strains and microbial consortia that provide robust pathogen defense without causing dysbiosis.

### Species-specific mechanisms of key beneficial bacteria

While the preceding sections describe general mechanisms employed by the microbiome, specific beneficial bacterial species exhibit distinct molecular strategies for suppressing pathogen virulence. This section examines the species-specific mechanisms of four key beneficial taxa: *Lactobacillus* spp., *Bifidobacterium* spp., *Bacteroides thetaiotaomicron*, and *Faecalibacterium prausnitzii*.

#### Lactobacillus species

*Lactobacillus* species are among the most extensively studied beneficial bacteria, with well-documented effects against multiple pathogens. Key mechanisms include:

*Lactic Acid Production and pH Reduction: Lactobacillus* spp. ferment carbohydrates to produce lactic acid, lowering local pH to levels (pH 4–5) that inhibit growth of acid-sensitive pathogens including *Salmonella*, *E. coli*, and *Staphylococcus aureus*. This acidification also enhances intestinal barrier function and modulates host immune responses [26].

*Bacteriocin Production*: Many *Lactobacillus* strains produce bacteriocins with narrow-spectrum activity against Gram-positive pathogens. For example, *Lactobacillus plantarum* produces plantaricin, which inhibits *Listeria monocytogenes* and *Clostridium* species by disrupting membrane integrity. These bacteriocins provide targeted pathogen defense while sparing Gram-negative commensals [26].

*Competitive Adhesion and Co-Aggregation: Lactobacillus* spp. competitively bind to intestinal epithelial cells and mucus, physically excluding pathogens from colonization sites. Additionally, certain *Lactobacillus* strains co-aggregate with pathogens such as *E. coli* and *Salmonella*, trapping them in the lumen and facilitating their clearance through peristalsis [26].

*Quorum Sensing Interference: Lactobacillus* species produce metabolites that interfere with AHL-based quorum sensing in Gram-negative pathogens, reducing virulence factor expression in *Pseudomonas aeruginosa*, *Vibrio cholerae*, and enterohemorrhagic *E. coli* [12].

*Immune Modulation: Lactobacillus* strains stimulate production of anti-inflammatory cytokines (IL-10, TGF-β) and promote regulatory T cell differentiation, creating an immune environment that tolerates commensals while maintaining responsiveness to pathogens. This immunomodulation is particularly important in preventing excessive inflammation during enteric infections [26].

#### Bifidobacterium species

*Bifidobacterium* species are dominant members of the infant gut microbiome and remain important throughout life. Their protective mechanisms include:

*Acetate Production: Bifidobacterium* spp. are major producers of acetate through fermentation of complex carbohydrates and human milk oligosaccharides. Acetate lowers intestinal pH, inhibits pathogen growth, and serves as a substrate for butyrate-producing commensals, indirectly enhancing colonization resistance. Acetate also strengthens epithelial barrier function by promoting tight junction assembly [25].

*Antimicrobial Metabolite Secretion: Bifidobacterium* strains secrete cell-free metabolites that inhibit pathogen growth and modulate commensal communities. These metabolites include organic acids, hydrogen peroxide, and uncharacterized antimicrobial compounds. Experimental studies show that *Bifidobacterium* cell-free extracts inhibit *Clostridioides difficile* growth and toxin production in vitro [25].

*Competitive Exclusion and Niche Occupation: Bifidobacterium* spp. efficiently colonize the intestinal mucosa, occupying niches that pathogens might otherwise exploit. Their ability to metabolize diverse glycans, including host-derived mucin oligosaccharides, allows them to thrive in the mucus layer and form a protective barrier against pathogen invasion [25].

*Immune Modulation and Barrier Enhancement: Bifidobacterium* strains stimulate secretory IgA production, enhance mucus secretion by goblet cells, and promote epithelial cell proliferation and repair. These effects collectively strengthen intestinal barrier function and reduce pathogen translocation. *Bifidobacterium* also modulates dendritic cell function, promoting tolerogenic immune responses that prevent excessive inflammation [25].

#### Bacteroides thetaiotaomicron

*Bacteroides thetaiotaomicron* is a prominent member of the human gut microbiome with sophisticated mechanisms for regulating pathogen virulence:

*Outer Membrane Vesicle (OMV) Production: B. thetaiotaomicron* produces outer membrane vesicles that modulate pathogen virulence through multiple mechanisms. These OMVs contain enzymes, lipopolysaccharides, and other bioactive molecules that interfere with pathogen adhesion, invasion, and toxin production. For example, *B. thetaiotaomicron* OMVs reduce *Shigella flexneri* virulence by interfering with the pathogen’s type III secretion system and preventing epithelial cell invasion [27].

*Polysaccharide Utilization and Niche Competition: B. thetaiotaomicron* possesses an extensive repertoire of polysaccharide utilization loci (PULs) that enable it to metabolize diverse dietary and host-derived glycans. This metabolic versatility allows *B. thetaiotaomicron* to dominate nutrient niches and outcompete pathogens for carbohydrate resources. By consuming glycans in the mucus layer, *B. thetaiotaomicron* also limits pathogen access to this colonization site [7].

*Immune Modulation: B. thetaiotaomicron* modulates host immune responses through production of polysaccharide A (PSA), a capsular polysaccharide that promotes regulatory T cell differentiation and IL-10 production. This immunomodulation maintains intestinal homeostasis and prevents excessive inflammation during pathogen challenge, indirectly limiting pathogen-induced tissue damage [4].

*Metabolic Cross-Feeding: B. thetaiotaomicron* engages in extensive metabolic cross-feeding with other commensals, producing acetate and other metabolites that support butyrate-producing bacteria and other beneficial taxa. These cross-feeding networks stabilize the microbiome and enhance colonization resistance against pathogens [7].

#### Faecalibacterium prausnitzii

*Faecalibacterium prausnitzii* is one of the most abundant butyrate-producing bacteria in the healthy human gut and is strongly associated with intestinal health and colonization resistance:

*Butyrate Production: F. prausnitzii* is a major producer of butyrate through fermentation of dietary fiber and acetate (produced by other commensals). Butyrate serves as the primary energy source for colonocytes, enhances epithelial barrier function, and exerts anti-inflammatory effects by inhibiting NF-κB signaling in immune cells. Butyrate also directly suppresses virulence gene expression in enterohemorrhagic *E. coli* and other pathogens [28].

*Anti-Inflammatory Metabolite Secretion*: Beyond butyrate, *F. prausnitzii* secretes other anti-inflammatory metabolites, including microbial anti-inflammatory molecule (MAM), which blocks NF-κB activation in intestinal epithelial cells and reduces pro-inflammatory cytokine production. This anti-inflammatory activity prevents the excessive inflammation that can promote pathogen expansion and tissue damage during infection [28].

*Colonization Resistance through Metabolic Niche Occupation: F. prausnitzii* occupies metabolic niches associated with fiber fermentation and acetate consumption, limiting the availability of these substrates for pathogens. Its abundance in the healthy gut correlates inversely with the presence of opportunistic Enterobacteriaceae, suggesting that *F. prausnitzii* contributes to colonization resistance through competitive exclusion [7].

*Immune Modulation and Barrier Enhancement: F. prausnitzii* promotes regulatory T cell differentiation and IL-10 production, contributing to intestinal immune homeostasis. It also enhances tight junction integrity and mucus production, strengthening the epithelial barrier against pathogen invasion. Reduced abundance of *F. prausnitzii* is associated with increased susceptibility to enteric infections and inflammatory bowel disease [28].

#### Summary of species-specific mechanisms

Table 1 (below) summarizes the key metabolites, producing bacteria, target pathogens, and virulence effects for the major mechanisms discussed in this section. These species-specific mechanisms highlight the functional diversity of the microbiome and underscore the importance of maintaining or restoring populations of key beneficial bacteria to achieve robust colonization resistance against pathogens.

**Table 1.** Key metabolites, producing bacteria, target pathogens, and virulence effects.

Metabolite/Mechanism	Producing Bacteria	Target Pathogen(s)	Virulence Effect	References
Short-Chain Fatty Acids (SCFAs)				
Butyrate	Faecalibacterium prausnitzii, Roseburia spp., Eubacterium spp.	Enterohemorrhagic E. coli (EHEC), Salmonella spp.	Downregulates virulence gene expression; enhances epithelial barrier function; anti-inflammatory effects	[20,28]
Propionate	Bacteroides spp., Phascolarctobacterium spp.	Salmonella spp., C. difficile	Modulates pathogen metabolism; reduces toxin production	[20]
Acetate	Bifidobacterium spp., Bacteroides spp.	Enterobacteriaceae, C. difficile	Lowers intestinal pH; substrate for butyrate producers; enhances barrier function	[20,25]
Bile Acids				
Secondary bile acids (e.g. deoxycholate, lithocholate)	Clostridium spp., Eubacterium spp. (via 7α-dehydroxylation)	Clostridioides difficile	Inhibits spore germination and vegetative growth; reduces toxin production	[21]
Lactic Acid				
Lactic acid	Lactobacillus spp., Bifidobacterium spp.	Salmonella spp., E. coli, S. aureus	Lowers pH; direct antimicrobial activity; inhibits pathogen growth	[26]
Bacteriocins				
Plantaricin	Lactobacillus plantarum	Listeria monocytogenes, Clostridium spp.	Disrupts membrane integrity; direct killing	[26]
Nisin	Lactococcus lactis	Staphylococcus aureus, Clostridium spp.	Pore formation in cell membrane; direct killing	[23]
Microcins				
Microcin B17 (MccB17)	E. coli (commensal strains)	Pathogenic Enterobacteriaceae	Inhibits DNA gyrase; blocks DNA replication	[24]
Microcin J25 (MccJ25)	E. coli (commensal strains)	Pathogenic Enterobacteriaceae, Salmonella spp.	Inhibits RNA polymerase; blocks transcription	[24]
Quorum Sensing Interference				
AHL-degrading enzymes (lactonases, acylases)	Bacillus spp., Rhodococcus spp., Bacteroides spp.	Pseudomonas aeruginosa, Vibrio cholerae, EHEC	Degrades autoinducers; prevents QS-dependent virulence activation	[13]
QS antagonists	Lactobacillus spp., probiotic yeasts	P. aeruginosa, S. aureus	Competitive inhibition of QS receptors; reduces virulence factor expression	[12,19]
Outer Membrane Vesicles (OMVs)				
OMVs	Bacteroides thetaiotaomicron	Shigella flexneri	Interferes with type III secretion system; prevents epithelial invasion	[27]
Hydrogen Peroxide				
H_2_O_2_	Lactobacillus spp., Streptococcus spp.	S. aureus, Streptococcus pyogenes	Oxidative stress; direct antimicrobial activity	[23]
Indole Derivatives				
Indole, indole-3-acetic acid	E. coli (commensal), Bacteroides spp.	Salmonella spp., pathogenic E. coli	Modulates virulence gene expression; affects motility and invasion	[20]
Immune Modulators				
Polysaccharide A (PSA)	Bacteroides fragilis, B. thetaiotaomicron	Indirect (via host immune modulation)	Promotes Treg differentiation; anti-inflammatory; enhances colonization resistance	[4]
Microbial anti-inflammatory molecule (MAM)	Faecalibacterium prausnitzii	Indirect (via host immune modulation)	Inhibits NF-κB; reduces pro-inflammatory cytokines; prevents pathogen-induced inflammation	[28]

## Clinical applications and therapeutic strategies

The mechanistic understanding of microbiome-mediated pathogen regulation has enabled development of novel therapeutic strategies that harness the protective capacity of commensal bacteria. These approaches represent a paradigm shift from pathogen-centric antimicrobial therapy toward ecosystem-based interventions that restore or enhance colonization resistance.

### Rationally designed probiotics

Traditional probiotics, typically consisting of *Lactobacillus* and *Bifidobacterium* strains, have been used empirically for decades with variable efficacy. Recent advances enable rational design of probiotic strains with specific anti-virulence properties tailored to target pathogens.

#### Mechanism-based strain selection

Modern probiotic development begins with identification of strains that exhibit specific mechanisms of pathogen inhibition. For example, strains that produce quorum quenching enzymes can be selected for their ability to interfere with QS-dependent virulence in *Pseudomonas aeruginosa* or *Staphylococcus aureus*. Similarly, strains that produce specific bacteriocins or metabolites can be selected based on their activity against target pathogens [19].

#### Genetic engineering for enhanced function

Synthetic biology approaches enable engineering of probiotic strains with enhanced anti-virulence properties. This includes overexpression of quorum quenching enzymes, introduction of novel bacteriocin production pathways, or optimization of metabolite production. Genetically edited probiotic consortia have been developed to modulate *Clostridioides difficile* virulence through enhanced production of specific metabolites that inhibit toxin production and spore germination [29].

#### Combination probiotic consortia

Rather than single-strain probiotics, rationally designed consortia that combine multiple strains with complementary mechanisms may provide more robust pathogen inhibition. These consortia can be designed to target multiple virulence pathways simultaneously, reducing the likelihood of pathogen adaptation or resistance. Consortium design is informed by ecological principles and systems biology modeling to ensure strain compatibility and functional synergy [29].

### Fecal microbiota transplantation (FMT)

FMT involves transfer of fecal material from a healthy donor to a recipient, with the goal of restoring a healthy, diverse microbiome that provides colonization resistance. FMT has proven highly effective for recurrent *Clostridioides difficile* infection, with cure rates exceeding 90% in many studies.

#### Mechanisms of FMT efficacy

FMT restores colonization resistance through multiple mechanisms: reestablishment of diverse commensal communities that compete with pathogens for nutrients and niches, restoration of secondary bile acid production that inhibits *C. difficile* germination, and normalization of immune function. The success of FMT demonstrates the power of ecosystem-level interventions for pathogen control [21].

#### Challenges and refinement

Despite its efficacy, FMT faces challenges including donor variability, risk of pathogen transmission, and lack of standardization. Current research focuses on identifying the minimal microbial consortia necessary for colonization resistance, enabling development of defined microbial therapeutics that provide FMT-like efficacy with improved safety and consistency. Several defined consortia are now in clinical trials for prevention of recurrent *C. difficile* infection and other indications [20].

### Precision microbiome engineering

Precision microbiome engineering represents the next frontier in microbiome-based therapeutics, leveraging detailed mechanistic knowledge and advanced technologies to design personalized interventions.

#### Personalized microbiome profiling

High-resolution metagenomic and metabolomic profiling of individual patients enables identification of specific microbiome deficits that increase pathogen susceptibility. For example, patients lacking key butyrate-producing bacteria or exhibiting reduced SCFA production may benefit from targeted supplementation with these functional groups. Personalized profiling also identifies patients most likely to benefit from specific interventions [20].

#### Targeted microbiome modulation

Rather than broad-spectrum interventions, precision approaches target specific microbial functions or taxa. This may involve selective depletion of pathogen-promoting bacteria, enrichment of protective commensals, or modulation of specific metabolic pathways. Dietary interventions, prebiotics, and narrow-spectrum antimicrobials can be used to achieve targeted modulation with minimal disruption of the broader microbiome [20].

#### Integration with conventional therapies

Microbiome-based interventions can be integrated with conventional antimicrobial therapy to improve outcomes and reduce recurrence. For example, administration of protective commensals during or immediately after antibiotic treatment may prevent secondary infections such as *C. difficile* by rapidly reestablishing colonization resistance. This integrated approach recognizes that pathogen eradication and microbiome restoration are complementary goals [20].

### Challenges and future directions for clinical translation

Despite promising advances, several challenges must be addressed to fully realize the clinical potential of microbiome-based therapeutics:

#### Inter-individual variability

Microbiome composition and function vary substantially among individuals, influenced by genetics, diet, environment, and prior exposures. This variability complicates development of universal interventions and necessitates personalized approaches. Better understanding of the factors driving inter-individual variation and identification of core functional modules that are conserved across individuals will facilitate more robust therapeutic design [20].

#### Regulatory and manufacturing challenges

Live biotherapeutic products face unique regulatory challenges related to product characterization, quality control, and safety assessment. Manufacturing of defined microbial consortia at scale while maintaining strain viability and functional activity requires specialized expertise and infrastructure. Regulatory frameworks are evolving to accommodate these novel therapeutics, but standardization remains a challenge [20].

#### Mechanistic understanding

While significant progress has been made, many aspects of microbiome-pathogen interactions remain incompletely understood. Deeper mechanistic knowledge is needed to predict intervention outcomes, optimize strain selection and consortium design, and identify biomarkers of treatment response. Continued investment in basic research using advanced model systems and multi-omics approaches will be essential [20].

## Conclusions

The human microbiome plays a critical and multifaceted role in regulating pathogen virulence and determining infection outcomes. Through mechanisms including quorum sensing interference, metabolite-mediated regulation, competitive exclusion, immune modulation, and direct antimicrobial production, commensal bacteria provide robust colonization resistance that operates within a complex triangular interaction involving host, microbiome, and pathogen. Key beneficial species—*Lactobacillus* spp., *Bifidobacterium* spp., *Bacteroides thetaiotaomicron*, and *Faecalibacterium prausnitzii*—employ distinct molecular strategies to suppress virulence of major pathogens including *Vibrio cholerae*, enterohemorrhagic *E. coli*, *Salmonella* spp., *Clostridioides difficile*, and *Staphylococcus aureus*.

Recent technological advances, particularly organ-on-chip platforms and multi-omics approaches, have transformed our ability to study these interactions with unprecedented resolution and physiological relevance, moving the field from correlation to causation. These tools have revealed the species-specific and context-dependent nature of microbiome-mediated pathogen regulation, highlighting the importance of community-level interactions and ecological principles.

Clinical translation of this knowledge has yielded promising therapeutic strategies, including rationally designed probiotics, fecal microbiota transplantation, and precision microbiome engineering. These ecosystem-based approaches represent a paradigm shift in infectious disease management, complementing traditional antimicrobial therapy with interventions that restore or enhance the protective capacity of the microbiome. The success of FMT for recurrent *C. difficile* infection demonstrates the clinical potential of microbiome-based therapeutics, while ongoing development of defined microbial consortia promises improved safety, consistency, and efficacy.

However, significant challenges remain, including inter-individual microbiome variability, incomplete mechanistic understanding of many microbiome-pathogen interactions, and regulatory and manufacturing hurdles for live biotherapeutic products. Addressing these challenges will require continued interdisciplinary collaboration among microbiologists, immunologists, clinicians, engineers, and regulatory scientists.

Ultimately, the recognition that infection outcomes are determined not by host-pathogen interactions alone, but by complex three-way interactions involving the resident microbiome, fundamentally changes how we approach infectious disease prevention and treatment. By harnessing the protective capacity of commensal bacteria, we can develop more effective, sustainable, and personalized strategies to combat infectious diseases in an era of rising antimicrobial resistance.

## Future directions

Looking forward, several key research priorities and technological developments will shape the next decade of microbiome-pathogen research and clinical translation:

### Advanced model systems

Next-generation organ-on-chip platforms that integrate multiple organ systems (gut-liver-immune chips) will enable study of systemic effects of microbiome-pathogen interactions and facilitate testing of interventions in physiologically realistic contexts. Integration of patient-derived cells and microbiomes in these platforms will support personalized medicine approaches and prediction of individual treatment responses.

### Artificial intelligence and machine learning

AI-driven analysis of large-scale multi-omics datasets will identify novel biomarkers of colonization resistance, predict infection risk based on microbiome profiles, and optimize design of therapeutic microbial consortia. Machine learning models trained on clinical cohorts and experimental data will enable rational selection of probiotic strains and personalized intervention strategies tailored to individual microbiome compositions.

### Expanded pathogen coverage

While current research has focused primarily on gastrointestinal pathogens, future work should expand to respiratory, urogenital, and skin pathogens, exploring how microbiomes at these sites regulate pathogen virulence. Understanding site-specific mechanisms and identifying protective commensals for non-gut niches will broaden the therapeutic applications of microbiome-based interventions.

### Phage-bacteria-host interactions

The role of bacteriophages in regulating both commensal and pathogen populations is increasingly recognized but remains understudied. Future research should investigate how phages modulate microbiome composition and function, influence pathogen virulence through horizontal gene transfer, and can be harnessed therapeutically in combination with bacterial probiotics.

### Microbiome-antimicrobial synergy

Rather than viewing microbiome-based interventions as alternatives to conventional antimicrobials, future strategies should explore synergistic combinations that leverage the strengths of both approaches. For example, narrow-spectrum antimicrobials that selectively target pathogens while sparing key commensals, or sequential therapy that combines pathogen eradication with rapid microbiome restoration, may achieve superior outcomes compared to either approach alone.

### Preventive microbiome engineering

Beyond treatment of established infections, future applications may focus on preventive microbiome engineering to reduce infection risk in high-risk populations (e.g. immunocompromised patients, surgical patients, travelers to endemic areas). Prophylactic administration of protective microbial consortia or targeted microbiome modulation through diet and prebiotics could provide durable colonization resistance and reduce reliance on antimicrobial prophylaxis.

### Regulatory and ethical frameworks

As microbiome-based therapeutics advance toward widespread clinical use, development of appropriate regulatory frameworks, quality standards, and ethical guidelines will be essential. This includes standards for donor screening and product characterization in FMT, guidelines for genetic engineering of probiotic strains, and frameworks for equitable access to these novel therapies.

### Global health applications

Microbiome-based interventions hold particular promise for global health applications, including prevention of cholera, typhoid, and other enteric infections in resource-limited settings. Development of low-cost, shelf-stable probiotic formulations and identification of locally available dietary interventions that enhance colonization resistance could have substantial public health impact in regions with high infectious disease burden.

By pursuing these research directions and translational priorities, the field will continue to advance from mechanistic discovery toward practical, scalable interventions that harness the protective power of the human microbiome to prevent and treat infectious diseases.

Pathogen virulence and infection outcomes are determined by bidirectional interactions among three components: the host immune system, the resident microbiome, and the invading pathogen. The host immune system shapes microbiome composition through secretory IgA, antimicrobial peptides, and inflammatory mediators, while the microbiome modulates host immunity through metabolites (SCFAs, bile acids), microbial-associated molecular patterns (MAMPs), and immune cell interactions. The microbiome directly regulates pathogen behavior via quorum sensing interference, metabolite production, nutrient competition, and antimicrobial secretion, while pathogens can exploit or disrupt commensal communities. The host immune response targets pathogens through innate and adaptive mechanisms, and pathogens deploy virulence factors to evade host defenses. Arrows indicate major interaction pathways; bidirectional arrows emphasize reciprocal regulation. This triangular framework underscores that infection outcomes cannot be understood by studying host-pathogen interactions in isolation, as the resident microbiome is an integral determinant of virulence regulation and disease susceptibility.

Table 1 Legend: This table summarizes the major metabolites and mechanisms by which beneficial bacteria regulate pathogen virulence, including the producing bacteria, target pathogens, specific virulence effects, and key references. SCFAs, bile acids, and direct antimicrobials represent the most extensively characterized mechanisms, while quorum sensing interference and immune modulation provide additional layers of protection. Species-specific mechanisms employed by *Lactobacillus*, *Bifidobacterium*, *Bacteroides*, and *Faecalibacterium* are highlighted throughout.

## Data Availability

Data sharing is not applicable to this article because no datasets were generated or analyzed during the current study. This review is based exclusively on previously published literature.
